# α-methyltryptophan-mediated protection against diabetic nephropathy in *db/db* mice as studied with a metabolomics approach

**DOI:** 10.3389/fphar.2024.1463673

**Published:** 2025-01-20

**Authors:** Aimin Cai, Dingchao Shen, Qiushuang Xiong, Jie Ding, Yang Ding, Xinlu Lin, Lijia Chen, Qing Yao, Guangyong Lin, Ruijie Chen, Vadivel Ganapathy, Longfa Kou

**Affiliations:** ^1^ Wenzhou Municipal Key Laboratory of Pediatric Pharmacy, Department of Pharmacy, The Second Affiliated Hospital and Yuying Children’s Hospital of Wenzhou Medical University, Wenzhou, China; ^2^ School of Pharmaceutical Sciences, Wenzhou Medical University, Wenzhou, China; ^3^ Department of Cell Biology and Biochemistry, Texas Tech University Health Sciences Center, Lubbock, TX, United States

**Keywords:** α-methyltryptophan, diabetic nephropathy, mTOR, apoptosis, metabolomics

## Abstract

**Introduction:**

Diabetic nephropathy (DN), a major complication of diabetes, presents with poor clinical outcomes and affects patients throughout their lifetime. α-Methyltryptophan (α-MT) is a blocker of the amino acid transporter. SLC6A14 and also an inhibitor of indoleamine 2,3-dioxygenase-1 (IDO1).

**Methods:**

In this study, we employed a nuclear magnetic resonance-based metabolomic approach to investigate the therapeutic effects of α-MT in a *db/db* mouse model of DN and explore the underlying molecular mechanisms.

**Results:**

The results of the study demonstrated that α-MT significantly reduced the urinary excretion of albumin and creatinine, improved kidney function, and decreased renal fibrosis in *db/db* mice. Metabolomic analyses of kidney tissues and urine samples indicated that *db/db* mice displayed increased activity of the enzyme IDO1, and alongside pronounced metabolic disturbances. These disturbances are chiefly characterized by alterations in amino acid metabolism, energy production pathways, membrane biochemical features, and nicotinamide metabolism, all of which have been implicated in mTOR signaling and apoptotic pathways.

**Discussion:**

Administration of α-MT to *db/db* mice showed evidence of IDO1 inhibition and rectification of metabolic dysfunctions with concurrent suppression of mTOR signaling and apoptosis. These findings highlight the potential of α-MT as a promising therapeutic agent for diabetic nephropathy.

## 1 Introduction

Diabetic nephropathy (DN) is the leading cause of end-stage renal disease which constitutes one of the major causes of death in both type 1 and type 2 diabetic patients (Chung et al., 2022). Despite continuous research and efforts directed at the early detection, prevention and treatment, the prevalence of DN has been rising continuously. Since 2011, the number of patients with chronic kidney disease related to diabetes has exceeded more than the number of patients with chronic kidney disease related to glomerulonephritis, and this difference keeps increasing in magnitude in China (Li et al., 2023). The first sign of kidney dysfunction of DN is microalbuminuria, which subsequently progresses in to macroalbuminuria, glomerulosclerosis, interstitial fibrosis, and end-stage renal disease (Thipsawat, 2021). It has been widely acknowledged that oxidative stress, inflammation and advanced glycation end products play a role in the pathogenesis of DN (Sun et al., 2024; Li et al., 2024). Apoptosis of glomerulus and activation of the mTOR pathway are also critical factors in DN (Zhong et al., 2023; Grahammer et al., 2021). Despite the availability of several treatments for DN, approximately half of patients with DN inevitably progress to end-stage renal disease (Gupta et al., 2023). As such, there is a pressing need for identification of new treatment strategies and drugs that are safe and effective for this debilitating disease.

α-MT is a well-established blocker of the amino acid transporter ATB^0,+^ encoded by the SLC6A14 gene. Pharmacological blockade of selective amino acid transporters in diabetes may limit availability of amino acids in the kidneys for protein synthesis/metabolism, thereby offering a potential avenue for the prevention and treatment of DN. Previous studies in this area have focused on several amino acid transporters, namely B0AT1 (SLC6A19) (Kano et al., 2024), LAT1 (SLC7A5) (Zhou et al., 2024), SNAT5 (SLC38A5) (Spears et al., 2023), vGLUT1 (SLC17A7) (Tian et al., 2024) and vGLUT2 (SLC17A6) (Zhang et al., 2024). Among these transporters, SLC6A19 and SLC7A5 are plasma membrane transporters for neutral amino acids, SLC38A5 is a plasma membrane transporter for amino acids such as glutamine, asparagine, histidine, serine, and glycine, and SLC17A6 and SLC17A7 are vesicular transporters for glutamate. Deletion of SLC6A19 in mice results in amino aciduria and also protects against diet-induced obesity, diabetes, and metabolic syndrome (Waters et al., 2021). This evidence suggests that amino acid transporters and the availability and metabolism of amino acids play a critical role in diet-induced obesity and diabetes. Furthermore, this implicates their involvement in DN. Most amino acid transporters have only a limited substrate selectivity, but ATB^0,+^ (SLC6A14) is a notable exception. ATB^0,+^ (SLC6A14) exhibits broad substrate specificity, facilitating the translocation of nearly all amino acids across the cell membrane, with the only exception of glutamate and aspartate (Anderson et al., 2022). α-MT, a well-studied blocker of this transporter, may facilitate restoration of impaired amino acid homeostasis in DN by modulating SLC6A14 activity. Obesity is considered an independent risk factor for the development of DN. In our recent study, α-MT was found to reduce food intake and body weight in multiple models of diet-induced obesity by a mechanism that is independent of its ability to block SLC6A14 (Sivaprakasam et al., 2021a). Moreover, α-MT is also an inhibitor of indoleamine-2,3-dioxygenase-1 (IDO1), which catalyzes the first step in the kynurenine pathway in tryptophan breakdown (Ganapathy, 2011). Elevated IDO1 activity and increased levels of kynurenine metabolites, which are activators of the AhR signaling pathway, are associated with inflammation and oxidative stress, processes implicated in the progression of DN (Yu et al., 2021). By targeting IDO1, α-MT may interfere with these detrimental mechanisms and ameliorate the pathological progression of the disease. In addition, the safety profile of α-MT has been established in numerous animal studies (Shoaf and Schmall, 1996; Sankoff and Sourkes, 1962). Thus, the known functions of α-MT in diverse biochemical processes involved in the pathogenesis and progression of DN underscore its potential as a promising therapeutic candidate for the alleviation of DN.

Nuclear magnetic resonance (NMR)-based metabolomics is a comprehensive method for monitoring metabolic changes in response to external stimuli. It provides valuable information for elucidating alterations in metabolic pathways that occur in an organism in response to such external stimuli (McCullagh and Probert, 2024). NMR, combined with multivariate statistics, has been applied extensively to explore the pathogenesis of various diseases and to study the consequences of various drugs (John et al., 2017; Shahisavandi et al., 2023). Metabolic disturbances have long been recognized as one of the primary underpinnings in the pathophysiology of DN (Han et al., 2024). Abnormal glucose metabolism is clearly a component of DN, but it represents only a part of the metabolic disturbances associated with this condition (Cheng et al., 2024). As a blocker of SLC6A14, IDO1 and metabolic syndrome, α-MT can potentially have profound impact on several downstream signaling pathways such as mTOR and AhR because decreased amino acid entry into cells would impair mTOR signaling and decreased generation of kynurenine would suppress AhR signaling. Even though the involvement of mTOR and AhR signaling pathways in the development of chronic kidney diseases has been well-established (Swaroop et al., 2024; Zhao et al., 2019), the potential of α-MT as a modulator of these pathways in the treatment of DN has not been investigated. Several studies have used the NMR-based metabolomics approach to investigate the impact of various therapies in DN (Tingting et al., 2020; Xiaodong et al., 2016). The goal of the present study was to evaluate the impact of α-MT treatment in DN in type 2 diabetic *db/db* mice using a ^1^H NMR-based metabolomics approach.

## 2 Materials and methods

### 2.1 Animal use and reagents

The *db/db* mice (C57BLKS/J-leprdb/leprdb, male) with an age of 8 weeks and their corresponding age-matched wild-type (WT, male) mice (provided by the Model Animal Research Center of Nanjing University, China) were raised in a temperature-controlled environment under an indoor temperature of 22°C ± 2°C, humidity of 50%–60%, and a 12 h/12 h light/dark cycle. The mice were provided with an adequate diet and access to drinking water throughout the duration of the experiment. Prior to the commencement of the experiment, all mice were maintained for a period of 1 week in accordance with standard practice.

α-Methyltryptophan (α-MT, Shenzhen Qimeike Biotechnology Co., Ltd.) was used as a SLC6A14 blocker in this study. The animals were randomly divided into four groups: wild-type control (WT), wild-type mice treated with α-MT (WT+ α-MT), *db/db* control (*db/db*), and *db/db* mice treated with α-MT (*db/db* + α-MT). α-MT was administered to mice intraperitoneally (i.p.) at a dose of 2.5 mg per mouse every other day for 8 weeks.

All procedures were conducted at the Experimental Animal Center of Wenzhou Medical University in accordance with the specifications of the Institutional Animal Care and Use Committee of WMU and the Declaration of Helsinki (as revised in 2013). The animal care and experimental protocols were approved by the Animal Ethics Committee of Wenzhou Medical University (approval number xmsq2022-1008).

### 2.2 Biochemical analysis and histopathological examination

The body weight and random blood glucose were monitored weekly throughout the experiment. Oral glucose tolerance test (OGTT) and insulin tolerance test (ITT) were performed on mice after a 12-h fasting, following 8 weeks of α-MT treatment. The levels of urinary albumin and urinary creatinine were determined using the urine protein quantitative test kit and Creatinine test kit (NanJing JianCheng Bioengineering Institute). For histopathological examination, the mice were euthanized using pentobarbital sodium, and kidneys were removed, fixed in 4% paraformaldehyde overnight, and processed for histological evaluation following the recommended protocol according to the Hematoxylin and Eosin Staining Kit (Shanghai Beyotime Bio-Technology Co. Ltd.).

### 2.3 Western blot analysis

Protein lysates were prepared from kidney tissues in RIPA lysis buffer, which was supplemented with a cocktail of protease inhibitors and a phosphatase inhibitor. Following the assessment of total protein concentration with the BCA Protein Assay Kit, 30 μg of protein were loaded and separated on 10% SDS–PAGE gels, and transferred at 80 V for an hour to PVDF membranes. The membranes were then incubated with 5% bovine serum albumin in Tris-buffered saline. The membranes were then incubated with primary antibodies for SLC6A14 (ab254786, Abcam), BCL-2 (ab182858, Abcam), BAX (2772S, CST), mTOR (2983s, CST) and phospho-mTOR (5536s, CST) at 4°C for 2 h. The membranes were then washed three times with TBST for 5 min each time. The membranes were then exposed to secondary antibodies (HRP-goat-anti-rabbit, cat. no. # ab6721, Abcam) for 2 hours at room temperature. The ECL plus Western blotting substrate (32132, Thermo Fisher Scientific) was employed to visualize the immunoreactive bands. The ImageQuant 5.2 software was employed to analyze the protein bands. The expression of β-actin was employed as a loading control, with the selective antibody (4970 S, Cell Signaling Technology) being used for this purpose.

### 2.4 Samples preparation and ^1^H NMR analysis

The mice were raised in metabolic cages for 24 h individually to collect urine samples. Urine samples were centrifuged for 10 min (3,000 g, 4°C) and the supernatants were extracted and stored at −80°C until NMR analysis. At 17 weeks of age, the animals were euthanized by cervical dislocation. The kidneys were removed, weighed, and put into liquid nitrogen immediately, and stored at −80°C.

200 μL of urine supernatants and 250 μL of phosphate buffer (pH 7.4) and 100 μL D_2_O containing sodium trimethylsilyl propionate-d4 (TSP, 0.1 mmol/mL) were mixed together to reduce changes in pH. The mixture was vortexed for 30 s on the ice, and centrifuged for 10 min (8,000 rpm, 4°C). 500 μL of the supernatants were extracted and transferred to NMR tubes for NMR detection. Kidney tissues were weighed and extracted with (4 mL/g) ice-cold methanol, ice-cold water (2.85 mL/g) and ice-cold chloroform (2 mL/g) using a tissue homogenizer for 90 s in an ice bath. After centrifugation (12,000 rpm, 10 min, 4°C), the upper liquid was lyophilized in a vacuum. The freeze-dried sample was dissolved in 550 μL D_2_O and centrifuged (12,000 rpm, 4°C) for 10 min 500 μL supernatants were transferred to NMR tube for analysis.

The high-resolution ^1^H-NMR spectra of urine and kidney extracts were obtained using the Bruker AVANCE III 600 spectrometer (Bruker BioSpin, Rheinstetten, Germany) equipped with a triple resonance probe. The spectra from the urine samples were collected using a one-dimensional 1D NOESY (noesypr1d) pulse sequence, and the spectra from the kidney samples were collected using CPMG (cpmgpr1d) sequence, both with 128 scans, 298 K, water suppression applied during the relaxation time for 6 s. The identification of metabolites was done using Chenomx NMR Suite (Edmonton, Canada), and the Human Metabolome Data Base (HMDB, http://www.hmdb.ca).

### 2.5 Multivariate pattern recognition analysis

Spectra were pre-processed using TopSpin 3.0 software (Bruker BioSpin, Rheinstetten, Germany) with manual phasing. Chemical shift calibrations were carried out relative to the methyl peak of lactate (CH3, 1.31 ppm) for urine and kidney samples. Matlab version R2012a (Mathworks, United Kingdom) and the “icoshift” procedure were used to analyze the NMR data. In Matlab, residual water signals (δ 4.70–5.00) and noise signals (regions before δ 0.5 and after δ 9.5) were excluded. The NMR regions were segmented and integrated into 0.01 ppm loading data for multivariate analysis and 0.0015 ppm loading data for quantitative analysis. Multivariate analysis was performed on Partial least squares-discriminant analysis (PLS-DA) that could discriminate metabolic differences among different groups by using SIMCA 12.0 software (Umetrics, Umeå, Sweden). The validity of the PLS-DA models was determined by the parameters R^2^, and the predictive capability was evaluated by Q^2^. The concentration of each metabolite was quantified according to its peak area by reference to the TSP concentration and was expressed as relative units (r.u.).

### 2.6 Statistical analysis

The statistical analysis was conducted using SPSS (version 16.0) software and one-way ANOVA. The data were expressed as mean ± standard deviation (means ± SD). The correlation analysis between tryptophan, kynurenine, kynurenine/tryptophan, and the mTOR/apoptosis pathway markers (Bax, Bcl-2, mTOR, pmTOR, Bax/Bcl-2, and pmTOR/mTOR) was performed using Spearman’s correlation on Chiplot Online (https://www.chiplot.online/). A p-value of less than 0.05 was considered to indicate a statistically significant difference.

## 3 Results

### 3.1 Protective effect of α-MT on diabetic nephropathy in *db/db* mice

**FIGURE 1:**
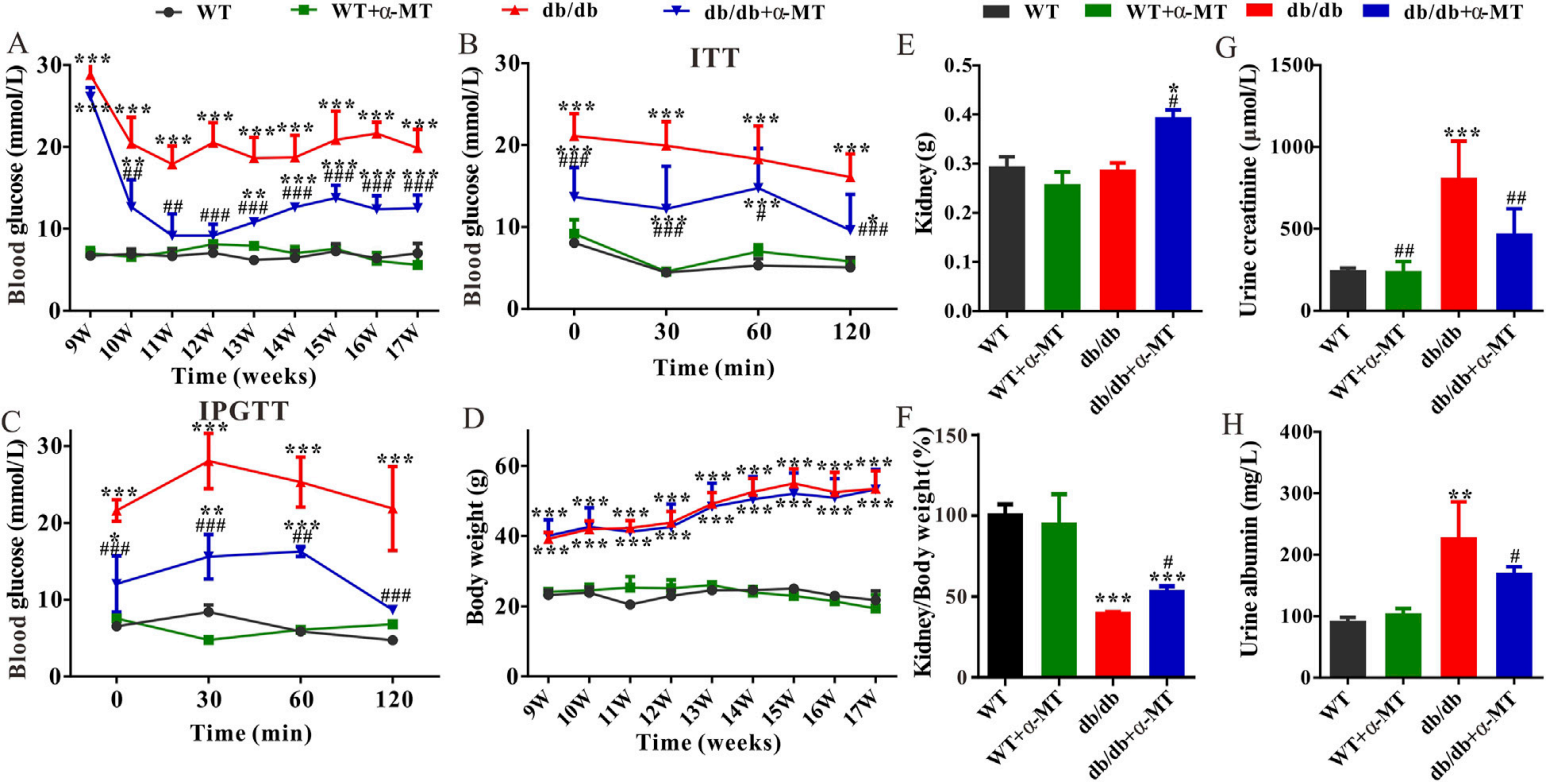
Effects of α-MT on biochemical features of diabetic nephropathy in *db*/*db* mice. **(A)** Blood glucose level; **(B)** Insulin tolerance test (ITT); **(C)** Intraperitoneal glucose tolerance test (IPGTT); **(D)** Body weight; **(E)** Kidney weight; **(F)** Ratio of kidney weight to bodyweight; **(G)** Urine creatinine level; **(H)** Urine albumin level. WT and *db*/*db* mice were treated with or without α-MT; the treatment began at 9 weeks of age and was continued until 17 weeks of age. *P < 0.05, **P < 0.01, and ***P < 0.001 versus WT mice; ^#^ *P* < 0.05, ^##^ *P* < 0.01, and ^###^ *P* < 0.001 versus *db*/*db* mice.

Compared with age-matched wild-type (WT) mice, the level of blood glucose in *db/db* mice was significantly increased at 9 weeks of age (Figure 1A). Following a one-week treatment with α-MT, the blood glucose levels of *db/db* mice exhibited a notable decline, whereas no discernible effect was observed in WT mice (Figure 1A). The inhibitory effect of α-MT on blood glucose levels in *db/db* mice was sustained throughout the treatment period, which lasted until the mice reached 17 weeks of age. The results of the ITT and IPGTT tests demonstrated that treatment with α-MT significantly enhanced insulin and glucose tolerance in *db/db* mice (Figures 1B, C). α-MT has previously been shown to effectively regulate body weight in obese mice (Sivaprakasam et al., 2021a). However, in our current experiment, the α-MT treatment had a negligible impact on the body weight of both *db/db* mice and wild-type (WT) mice (Figure 1D). This discrepancy may be attributed to differences in either the dosage of α-MT or mode of administration (oral versus intraperitoneal). Figure 1E illustrates that the weight of the kidney was significantly elevated in *db/db* mice subjected to α-MT treatment in comparison to control *db/db* mice. However, no discernible difference was observed in WT mice with and without α-MT treatment. The kidney-to-body weight ratio was significantly lower in control *db/db* mice compared to WT mice; following treatment with α-MT, this ratio increased in *db/db* mice (Figure 1F). α-MT treatment had no effect on this parameter in WT mice. The levels of creatinine and albumin in urine were measured for evaluation of renal function. The urinary excretion of creatinine and albumin in *db/db* mice was significantly higher than in age-matched WT mice. This increase was reversed significantly with α-MT treatment (Figures 1G, H). The treatment had no effect on this parameter in WT mice.

**FIGURE 2:**
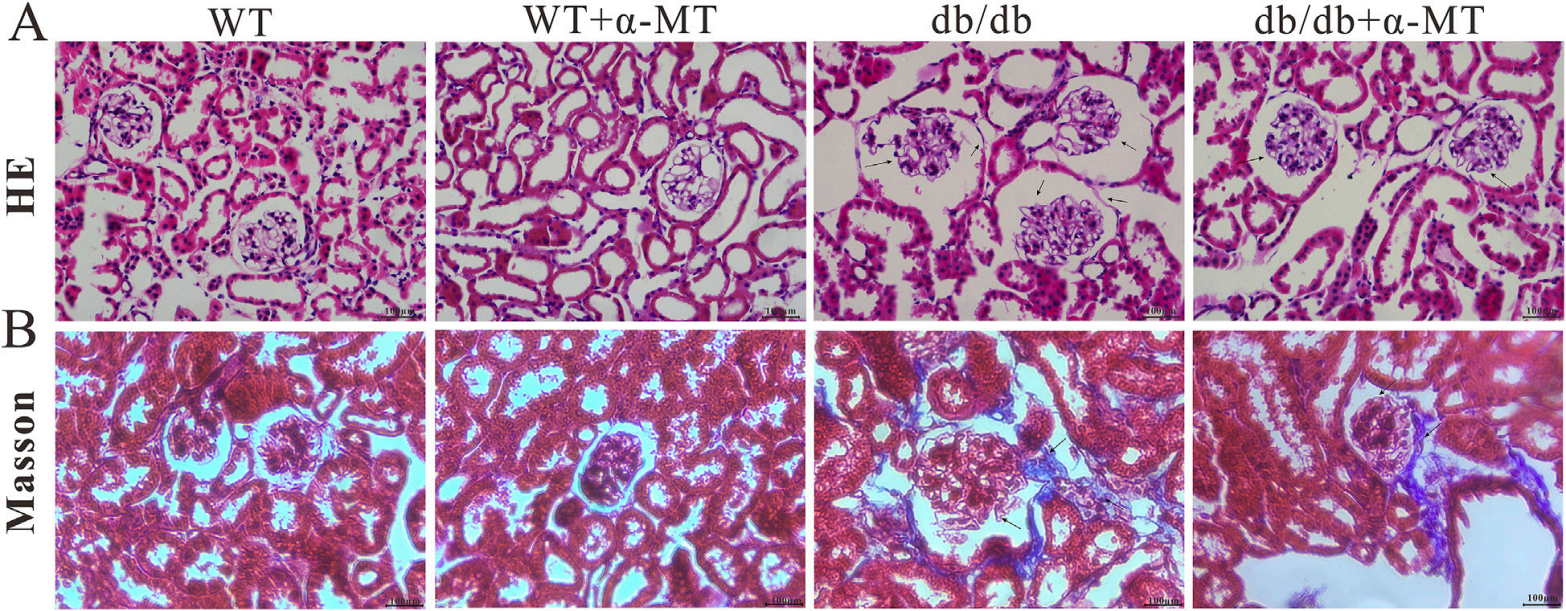
Representative images of renal tissue sections from 17-week-old WT and *db*/*db* mice with and without α-MT treatment: H & E staining **(A)**; Trichrome (Masson) staining **(B)**. The treatment began at 9 weeks of age and was continued until 17 weeks of age.

At the histological level, H&E staining revealed the pathological characteristics of DN, including glomerulus hypertrophy, mesangial matrix expansion, and basement membrane thickening (indicated by arrows). However, treatment with α-MT caused improvement in these parameters (Figure 2A). Furthermore, Masson’s trichrome staining demonstrated the presence of renal fibrosis (blue staining) in *db/db* mice; α-MT treatment resulted in a reduction of this staining, indicating protection against fibrosis (Figure 2B). The administration of α-MT to WT mice did not result in any discernible changes in the kidneys.

### 3.2 Metabolic profiles in kidney and urine in *db/db* mice treated with α-MT

The metabolite profiles of kidney and urine samples were analyzed using ^1^H-NMR in wild-type and *db/db* mice with and without α-MT treatment. Supplementary Figures S1A, B illustrate the typical ^1^H NMR spectra of the kidney and urine in *db/db* mice, respectively. The ^1^H-NMR-based metabolic profile identified a total of 37 metabolites, including amino acids (tryptophan, alanine, N-acetylglutamate, glutamate, glutamine, phenylalanine, methionine, taurine, tyrosine, valine, isoleucine, leucine), and energy metabolism-related metabolites (succinate, citrate, creatine, creatine phosphate, trans-aconitate, lactate, pyruvate, glucose). The other metabolites identified were: fumarate, ADP, AMP, inosine, choline, PC, GPC, 3-hydroxybutyrate, uridine, uracil, adenine, formate, acetate, dimethylamine, niacinamide, trigonelline, hippurate, benzoate, myo-inositol, 1-methylnicotinamide. The metabolites were identified in accordance with the Reference Library of the Chenomx NMR suite 7.0 (Chenomx Inc., Edmonton, Canada) and a public NMR database (Human Metabolome Database; www.hmdb.ca).

The differences in metabolomics profiles between the wild-type (WT), *db/db*, and their corresponding α-MT-treated groups were evaluated by Partial Least Squares-Discriminant Analysis (PLS-DA). In this method of analysis, each data point represents an independent sample, which is a summary of information from all molecules. Consequently, the distance between points is a measure of similarity in metabolomic profiles between different samples. The closer the points cluster together, the more similar the metabolomic profiles are. The PLS-DA score plots based on the kidney metabolome (Supplementay Figure S2A) and urine metabolome (Supplementay Figure S2B) both demonstrated clear differences among the WT, *db/db* and *db/db* treated with α-MT groups. Nevertheless, the WT group exhibited some degree of overlap with the α-MT-treated WT group, suggesting that α-MT exerted a minimal influence on the metabolomic profile of WT mice. Furthermore, the α-MT-treated *db/db* group exhibited a closer proximity to the WT group, suggesting that the metabolomic shift resulting from α-MT treatment is more closely aligned with the WT group than the untreated *db/db* group. A comparison of the four groups was conducted using PLS-DA models. The R2 parameter of the PLS-DA score plots provided evidence of good fitness, while the Q2 parameter indicated high predictability of the models. The R2 and Q2 values for the WT vs. *db/db* and *db/db* vs. *db/db* + α-MT models in the kidney and urine (Table 1) exceeded 0.4, indicating that the model in these groups is robust. Nevertheless, the Q2 values for WT vs. WT + α-MT were less than zero for both kidney and urine metabolomes, providing further evidence that α-MT had no discernible effect on WT mice. This also attests to the safety of the drug treatment. Concurrently, the PLS-DA plots demonstrate a clear indication of a substantial impact on the metabolomic profile in the kidneys and urine of *db/db* mice, resulting in a reversal of these phenotypes and a movement closer to those observed in WT mice. A significant discrimination was observed between the *db/db* group and the α-MT-treated *db/db* group, both in the kidney metabolome (Supplementay Figure S2C) and urine metabolome (Supplementay Figure S2D). The results demonstrate that the administration of α-MT to *db/db* mice had a significant impact on the metabolomic phenotype of DN.

**TABLE 1:** Comparison of parameters of PLS-DA for metabolites in kidney and urine between different groups.

Parameters	Kidney		Urine	
	R^2^	Q^2^	R^2^	Q^2^
*db*/*db* vs. WT	0.467	0.702	0.613	0.620
*db*/*db* vs. *db*/*db* + α-MT	0.501	0.584	0.706	0.857
WT vs. WT + α-MT	0.46	−0.087	0.88	−0.210

### 3.3 α-MT changes the metabolites in the kidney and urine of *db/db* mice

**FIGURE 3:**
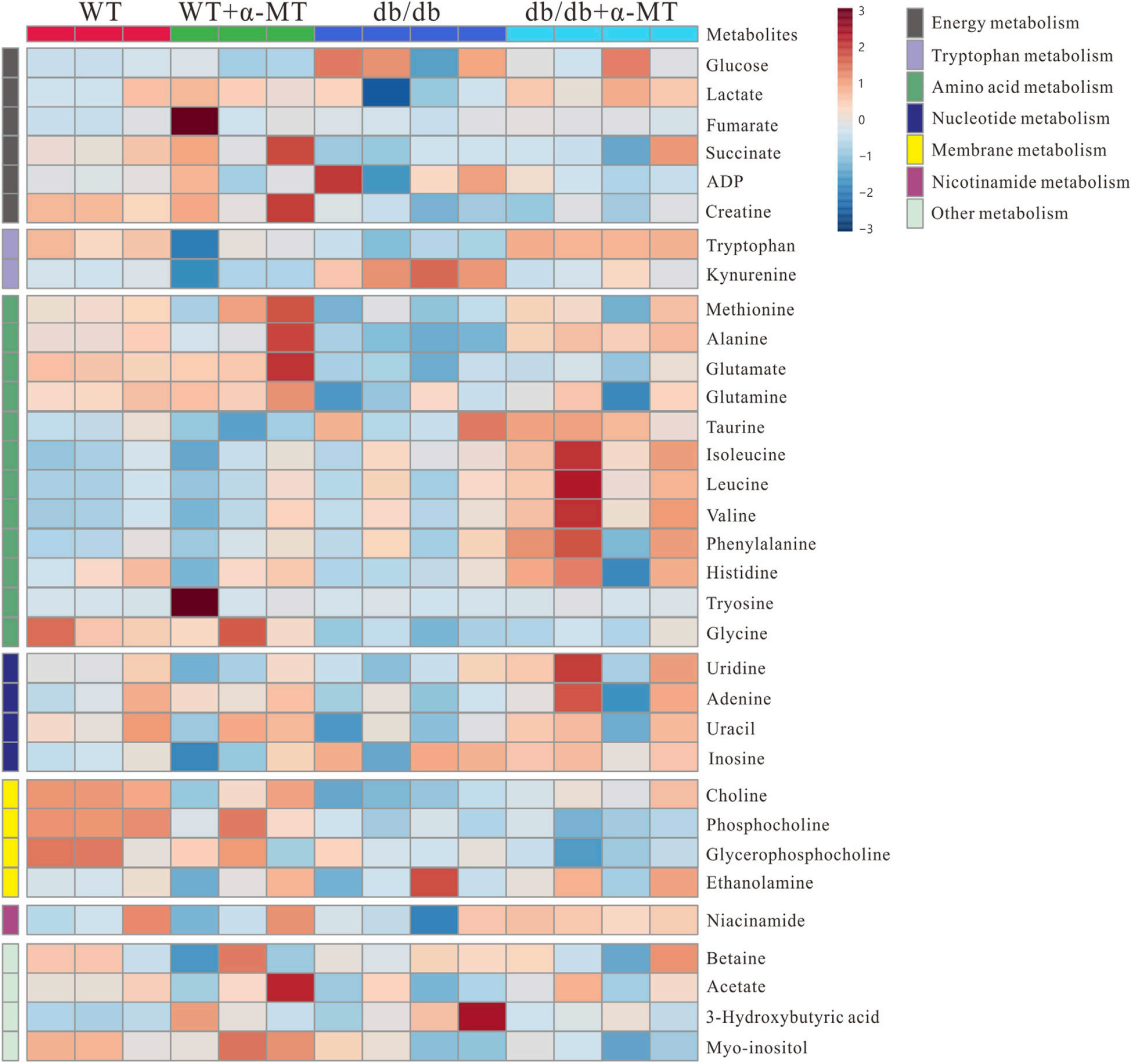
Heatmap for variations in metabolites in kidney tissues from the four groups of mice (WT, WT + α-MT; *db*/*db*, *db*/*db* + α-MT). The treatment with α-MT began at 9 weeks of age and was continued until 17 weeks of age. The shades of red (color scale > 0) represent higher concentration of metabolites, and shades of blue (color scale < 0) indicates lower concentration of metabolites.

**FIGURE 4:**
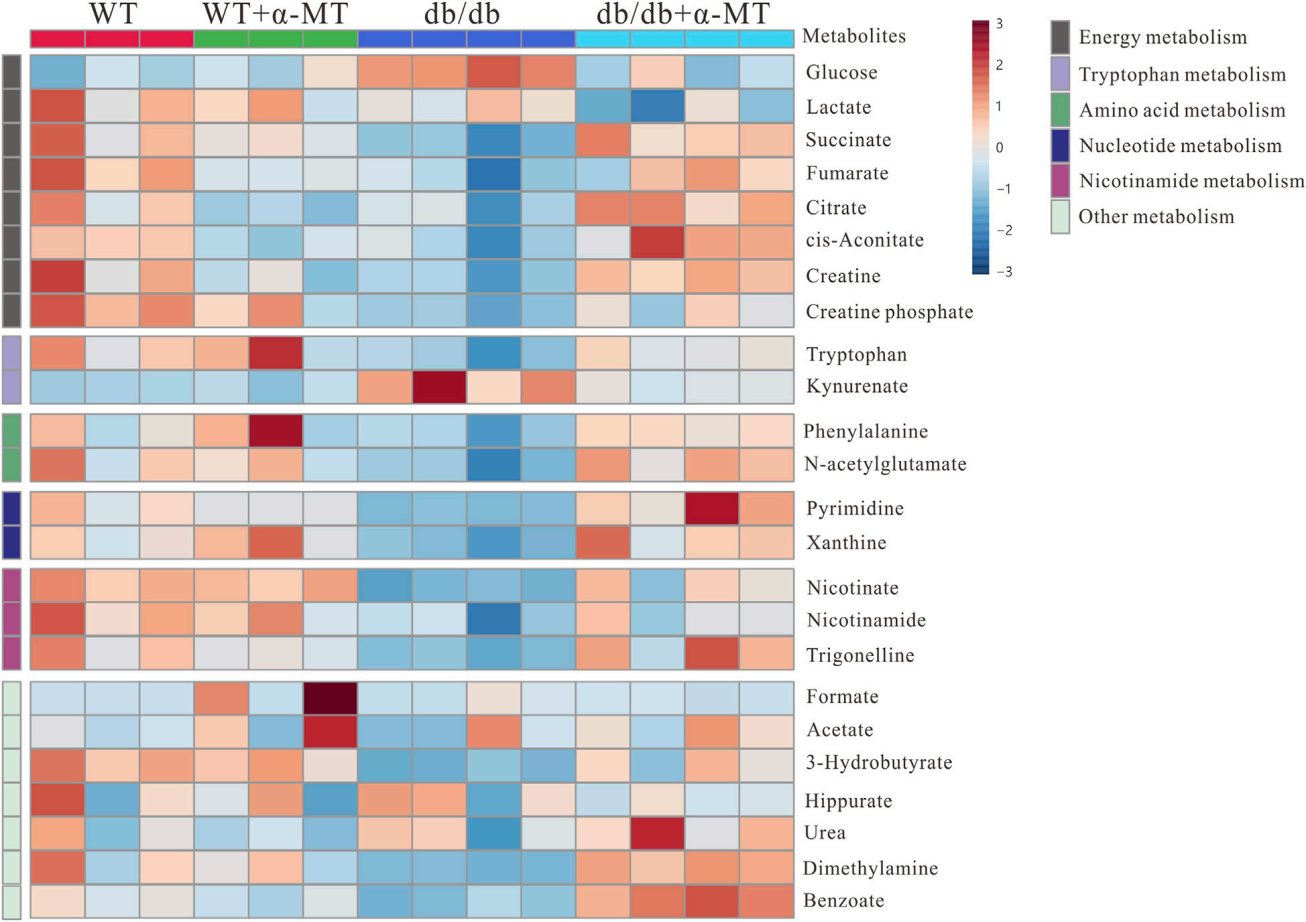
Heatmap for variations in metabolites in urine samples from the four groups of mice (WT, WT + α-MT; *db*/*db*, *db*/*db* + α-MT). The treatment with α-MT began at 9 weeks of age and was continued until 17 weeks of age. The shades of red (color scale > 0) represent higher concentration of metabolites, and shades of blue (color scale < 0) indicates lower concentration of metabolites.

**FIGURE 5:**
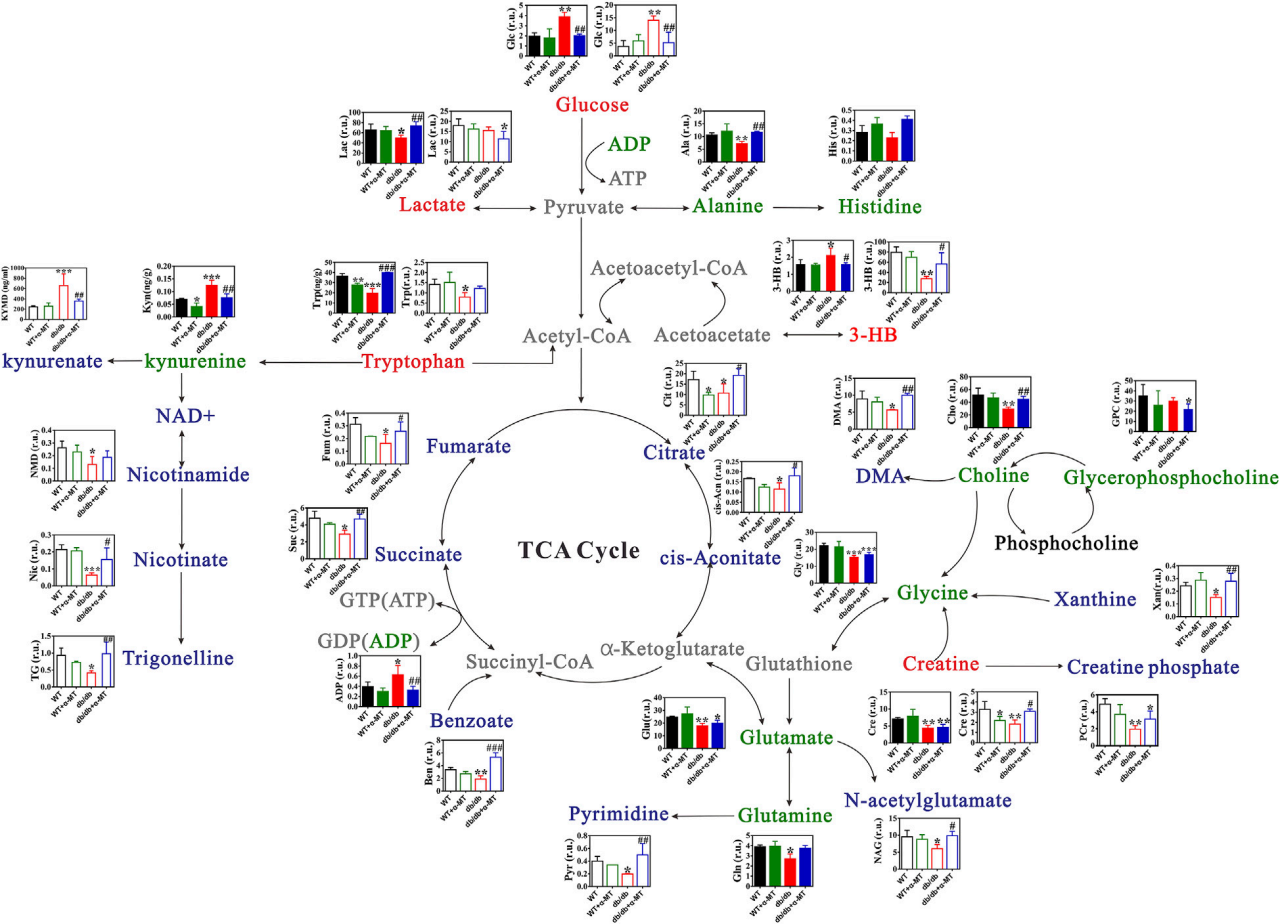
Changes in metabolite levels in kidney and urine of WT and *db*/*db* mice with and without α-MT treatment. Green texts and histogram with closed bars represent metabolites in kidney samples. Blue texts and histogram with open bars indicate metabolites in urine samples. ^#^ *P* < 0.05, ^*##*^ *P* < 0.01 and ^###^ *P* < 0.001 versus *db*/*db* mice; *P < 0.05, **P < 0.01, and ***P < 0.001 versus WT mice. 3-HB, 3-Hydroxybutyrate; DMA, Dimethylamine.

To gain further insight into the impact of α-MT on the DN, we employed quantitative and comparative analysis of specific metabolites derived from ^1^H-NMR data. Figures 3, 4 present the heatmaps of the identified metabolites from the kidney and urine, respectively. In the case of kidney metabolites, the *db/db* group exhibited an increase in the levels of glucose, kynurenine, 3-hydroxybutyrate, and ADP. These increases were significantly blunted in the α-MT-treated *db/db* group. Conversely, the *db/db* group exhibited significantly lower levels of creatine, methionine, alanine, glutamate, glutamine, tryptophan, choline and niacinamide than the WT group. However, α-MT treatment significantly reversed this effect. However, α-MT treatment in *db/db* mice resulted in increased levels of taurine, phenylalanine, histidine, isoleucine, leucine, and valine compared to WT mice. In the case of urinary metabolites, the concentration of glucose and kynurenate were increased in *db/db* mice and decreased in α-MT-treated *db/db* mice, in comparison to WT mice. In comparison to WT mice, *db/db* mice exhibited significantly reduced levels of succinate, fumarate, citrate, trans-aconitate, creatine, creatine phosphate, phenylalanine, N-acetyl glutamate, tryptophan, pyrimidine, xanthine, adenosine, 3-hydroxybutyrate, dimethylamine and benzoate. However, α-MT treatment resulted in elevated levels of these metabolites in *db/db* mice. Figure 5 provides a summary of the location of these metabolites within specific metabolic pathways, along with an illustration of the changes in their levels in the kidney and urine. The metabolites that exhibited notable alterations in response to α-MT treatment were associated with energy metabolism, amino acid metabolism, nucleic acid metabolism, and membrane lipid composition.

### 3.4 α-MT suppresses apoptosis and mTOR signaling in the kidneys of *db/db* mice

**FIGURE 6:**
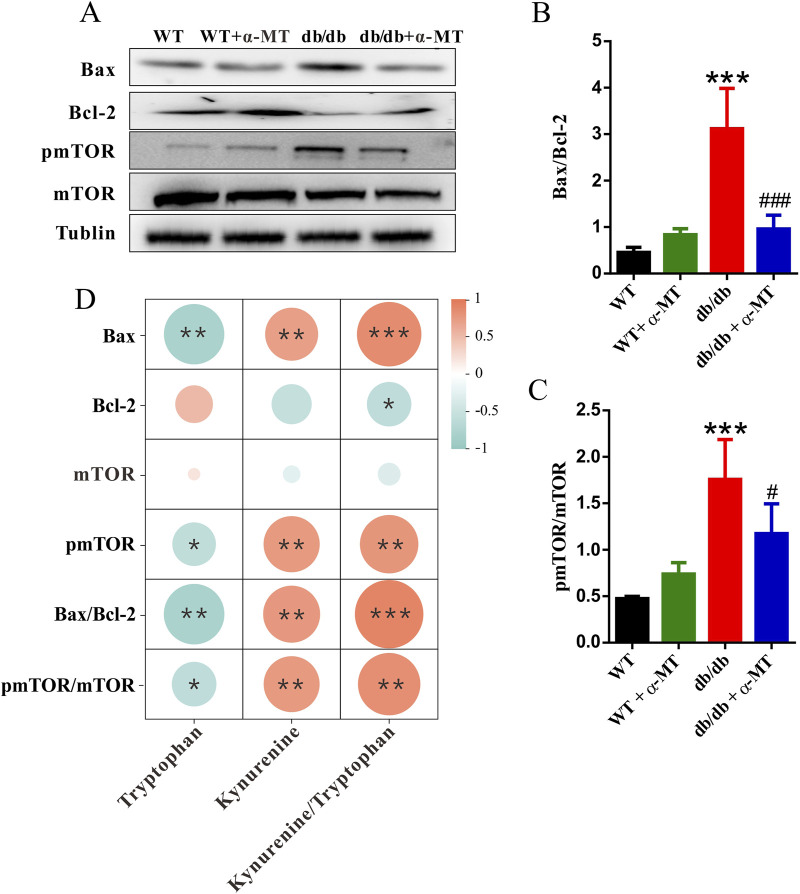
α-MT suppresses apoptosis and mTOR phosphorylation in kidneys of *db*/*db* mice. **(A)** Western blot analysis of Bax, Bcl-2, phospho-mTOR, mTOR, and Tubulin in renal tissues of WT and *db*/*db* mice with and without α-MT treatment. **(B)** Protein levels of Bax normalized to Bcl-2. **(C)** Levels of phospho-mTOR normalized to mTOR. **(D)** Heatmap of the correlation between tryptophan, kynurenine, kynurenine/tryptophan, and the mTOR/apoptosis pathway markers (Bax, Bcl-2, mTOR, pmTOR, Bax/Bcl-2, and pmTOR/mTOR) using Spearman’s correlation. The size of the circles represents the magnitude of the correlation. Blue and red indicate negative and positive correlations, respectively. *P < 0.05, **P < 0.01, and ***P < 0.001 compared to WT mice; ^#^ *P* < 0.05, ^##^ *P* < 0.01, and ^###^ *P* < 0.001 compared to *db*/*db* mice.

Compared to age-matched wild-type mice, the level of the anti-apoptotic protein Bcl-2 was significantly decreased, while the pro-apoptotic protein Bax was increased in the kidneys of *db/db* mice (Figure 6A). These data demonstrate the involvement of increased apoptotic cell death in the kidney tissues associated with DN in *db/db* mice. Treatment of the *db/db* mice with α-MT reversed these changes; the levels of Bcl-2 increased and those of Bax decreased as a result of the treatment. The effects of α-MT on the levels of these two proteins were minimal in WT mice. The quantification and the relative revels of the two proteins as Bax/Bcl-2 ratio are given in Figure 6B. Furthermore, the activity of the mTOR signaling pathway was monitored by examining the phosphorylation status of mTOR (Figure 6A). In *db/db* mice, the levels of phospho-mTOR and the ratio of phospho-mTOR/mTOR were significantly increased in comparison to WT mice (Figure 6C), indicating that the mTOR pathway is activated in diabetes-associated kidney dysfunction. Treatment with α-MT blunted this change in *db/db* mice. In WT mice, the effect of α-MT was minimal.

α-MT is known to inhibit IDO1. To elucidate the relationship between IDO1 and the mTOR/apoptosis pathway, we evaluated the correlations between tryptophan and its metabolite kynurenine in the kidney, as well as the kynurenine-to-tryptophan ratio (which reflects IDO1 activity) and the mTOR/apoptosis pathway using Spearman’s correlation test (Figure 6D). The results indicated that the expression levels of BAX, pmTOR, the Bax/Bcl-2 ratio, and the pmTOR/mTOR ratio were negatively correlated with tryptophan levels but positively correlated with kynurenine levels and the kynurenine-to-tryptophan ratio. This suggests a close association between IDO1 and the mTOR/apoptosis pathway.

**FIGURE 7:**
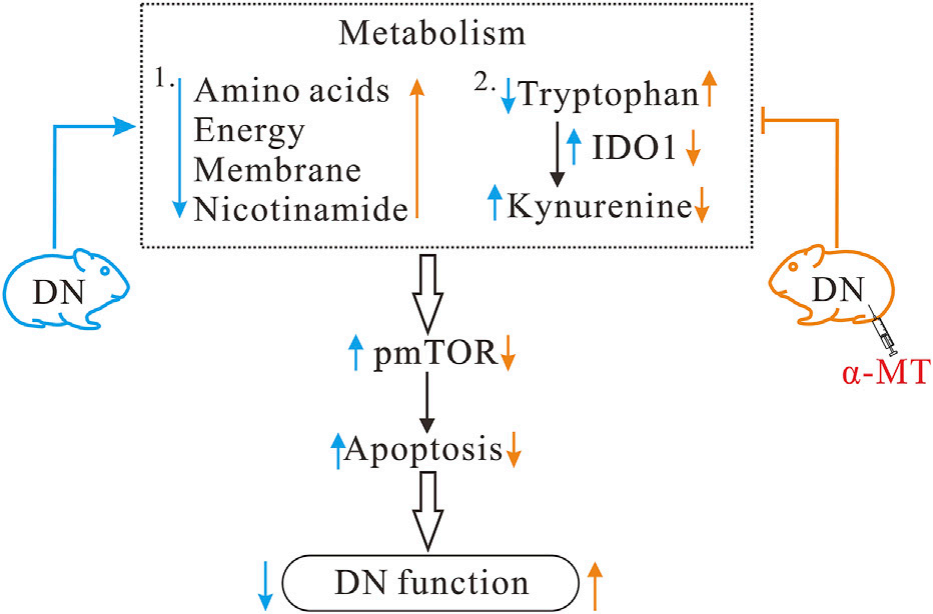
Potential mechanisms for the protective effect of α-MT in diabetic nephropathy. α-MT treatment corrected metabolic disturbances and suppressed mTOR signaling and apoptosis in *db*/*db* mice with diabetic nephropathy. Black arrow indicates changes in diabetic nephropathy and red arrow indicates changes after α-MT treatment.

As illustrated in Figure 7, the present study demonstrates that diabetic nephropathy is associated with significant alterations in specific metabolic pathways. Furthermore, the results indicate that treatment with α-MT is able to reverse these alterations to a significant extent. In addition to the changes in metabolic phenotype, diabetic nephropathy is also associated with increased apoptosis and increased mTOR, and possibly AhR, signaling. The administration of α-MT corrects the metabolic alterations and the resulting apoptotic process in the kidney that are characteristic of diabetic nephropathy.

## 4 Discussion

DN is one of the most lethal complications of diabetes, occurring in 25%–40% of diabetes patients (Liu et al., 2022). At present, no effective treatment regimen offers a cure for diabetic nephropathy (DN). Consequently, there is an urgent need for the identification of new targets to facilitate the management of this disease. The present study examined the influence of the tryptophan derivative α-MT on the pathogenesis and progression of DN in the *db/db* mouse model. α-MT is known to have a plethora of pharmacological effects, including the blockade of the amino acid transporter SLC6A14 (Lu et al., 2022; Cai et al., 2020; Karunakaran et al., 2011), inhibition of IDO1 (Ganapathy, 2011), and control of body weight (Sivaprakasam et al., 2021a). Since mTOR is a signaling pathway influenced by cellular amino acid status, this pathway is affected by the activity of the amino acid transporter SLC6A14. IDO1 generates kynurenine, an endogenous activator AhR, a signaling pathway with profound impact on inflammation and redox status of the cell. Published reports have shown therapeutic benefits of suppressing the mTOR and AhR signaling pathways in the treatment of kidney diseases, including DN (Swaroop et al., 2024; Zhao et al., 2019). As a blocker of SLC6A14 and an inhibitor of IDO1, α-MT is expected to suppress these signaling pathways in *db/db* mice with potential amelioration of DN. The results of the present study provide supporting evidence for this expectation as seen in terms of blood glucose and ketone body levels, ITT and IPGTT patterns, and histological features of kidney tissue sections. This is also clearly evident from the kidney function tests that focused on urinary excretion of albumin and creatinine. Based on these findings, we conclude that α-MT holds promise, either as a candidate drug or at least as a lead compound for future development of better drugs, for DN treatment.

To understand the molecular and biochemical bases of the observed beneficial effects in *db/db* mice, we used a metabolomic approach to determine potential changes in metabolic pathways associated with DN and to evaluate the impact of α-MT on such changes as a guide to determine its potential as a drug for the treatment of DN. First, we looked at the possible involvement of SLC6A14 blockade in the observed beneficial effects of α-MT. Tryptophan is a transportable substrate for SLC6A14 (Karunakaran et al., 2011). α-MT, a derivative of tryptophan, is not a transportable substrate for SLC6A14; instead, it is a blocker of the transporter (Karunakaran et al., 2008). SLC6A14 has received much attention for its role as a tumor promoter (Nałęcz, 2020). In addition, recent studies have demonstrated an important role for this transporter in obesity (Bhutia et al., 2022). Deletion of SLC6A14 in mice does not lead to significant detrimental consequences under normal conditions (Babu et al., 2015), even though the null mice are resistant to certain cancers (Babu et al., 2015; Schniers et al., 2022) and also are prone to obesity when fed a high-fat diet (Sivaprakasam et al., 2021b). Treatment of mice with α-MT is therefore expected to cause a decrease in the transport function of SLC6A14, but the observed protective effects of α-MT against DN seem counter-intuitive based on the published literature that SLC6A14 deficiency leads to diet-induced obesity, insulin resistance, diabetes, metabolic syndrome, and fatty liver. This suggests that the function of α-MT as a blocker of this amino acid transporter is most likely unrelated to the findings of the current study. The metabolomics of α-MT-treated *db/db* mice supports this view. The concentrations of several amino acids in kidneys of *db/db* mouse, including tryptophan, are reduced compared to WT mice; this decrease is blunted with α-MT treatment. The function of α-MT as a blocker of SLC6A14 does not provide any logical explanation for these findings. The expression of this transporter shows limited tissue distribution with its expression evident only in a small number of tissues such as the intestinal tract, lungs, and conjunctiva (Sikder et al., 2017; Bhutia et al., 2014). We examined the expression of this transporter in kidney and found no evidence for its expression in WT or *db/db* mouse kidneys (data not shown). The absence of SLC6A14 in kidney also shows that blockade of the transporter by α-MT could not have contributed to the observed changes in amino acids in this tissue in α-MT-treated *db/db* mice.

Kidney is one of the tissues that exhibits very high energy dependence, only next to brain, heart, and skeletal muscle. Earlier studies using the metabolomics approach showed progressively reduced tricarboxylic acid (TCA) cycle intermediates such as citrate, succinate, aconitate with age in *db/db* mice, indicating perturbations in the TCA cycle in association with the development of DN (Stec et al., 2015; Mengjie et al., 2013). The data from our current study corroborate these earlier findings. We found decreased levels of citrate, fumarate, succinate, and aconitate in *db/db* mice compared to WT mice. In addition, increased levels of glucose and decreased levels of lactate found in the kidneys of *db/db* mice is indicative of defective glycolysis, again contributing to the energy crisis in this tissue. Treatment with α-MT reverses these trends in energy metabolites in *db/db* mouse kidneys, thus indicating that α-MT improves the energy status of the kidney tissue. This could offer at least a partial biochemical explanation for the protection by α-MT against DN. The same is true with creatine and phosphocreatine, which are important mechanism for the storage of energy. Creatine is widely used as a nutritional supplement only based on its biological role in energy storage (Kazak and Cohen, 2020). It has been reported that phosphocreatine could improve mitochondrial function and protect against diabetes-induced kidney injury by reducing oxidative stress and apoptosis (Abdullah et al., 2020). Our results showed the level of creatine and phosphocreatine in *db/db* mice were significant decreased, compared with age-matched WT mice, but this change was recovered by α-MT treatment. This provides further evidence for the improvement of the energy status of the kidney tissue by α-MT.

The lipid composition of cell membranes involves the metabolism of glycerophosphocholine (GPC), phosphocholine (PC), and choline. DMA is produced from the degradation of choline by the gut microflora. In our research, the levels of choline and DMA in *db/db* mice were significantly lower than age-matched WT mice, but their levels were improved by α-MT treatment significantly, suggesting a recovery of membrane-lipid metabolism that is defective in diabetes. 3-HB is the principal ketone body, which has multiple biological functions, including as an energy substrate, as an inhibitor of histone deacetylases, and as an agonist for the cell-surface G-protein-coupled receptor GPR109A (Zhang et al., 2021). It also participates in post-translational modification of proteins via β-hydroxybutyrylation. The metabolomics analysis in the present study shows decreased levels of 3-HB in the urine samples and increased levels in kidney tissues in *db/db* mice, and these changes were reversed with α-MT treatment. Ketogenesis is the metabolic pathway that produces 3-HB; it occurs primarily in the liver, but kidney is also capable of ketogenesis (Nakatani et al., 1996). In diabetes, fatty acid oxidation and ketogenesis are accelerated with resultant 3-HB production. This metabolite functions as an energy source alternative to glucose in diabetes, and its generation is dependent on the availability of fatty acids as the fuel to β-oxidation and ketogenesis. The levels of fatty acids in the circulation are known to be elevated in diabetes, which are taken by the liver to feed into fatty acid oxidation.

Nicotinamide is a component of NAD^+^ and NADP^+^ that are critical for cell respiration and energy production. Niacinamide has other functions as well; it protects against apoptosis, inflammation and insulin resistance (Mihoko et al., 2021). It is also an inhibitor of the histone deacetylase SIRT1. It is reported that nicotinamide also prevents kidney injury (Meiling et al., 2019). Nicotinamide is also related to the vitamin nicotinic acid. The decreased levels of nicotinamide in *db/db* mouse kidneys might reflect deficiency of this vitamin. Nicotinic acid suppresses lipolysis in adipocytes via GPR109A, the key determinant of fatty acids in circulation (Lukasova et al., 2011). The possible decrease in nicotinic acid levels in *db/db* mice might be relevant to the increased circulating levels of fatty acids in these mice. Trigonelline (N-methyl nicotinic acid) is a major alkaloid in fenugreek, a traditional Chinese herb; approximately 85% of the trigonelline is metabolized in the body to yield nicotinate. Diets containing nicotinic acid and trigonelline tend to improve glucose tolerance (Gary et al., 2014). Trigonelline is beneficial in the prevention and treatment of diabetes and diabetic complications by inhibiting intestinal glucose uptake, promoting β cell regeneration and insulin secretion (Zhou et al., 2012). Li et al. reported that trigonelline reduced diabetic nephropathy and insulin resistance in type 2 diabetic rats (Yinyan et al., 2019). Our data show that *db/db* mice had lower level of nicotinamide, nicotinic acid and trigonelline than in age-matched WT mice, and α-MT treatment led to a significant increase in the levels of all three metabolites. It is possible that these changes contribute to the observed beneficial effects of α-MT in *db/db* mice in the form of protection against DN.

Approximately 95% of the tryptophan is degraded via the kynurenine pathway involving two key enzymes: tryptophan-2,3-dioxygenase (TDO) which is highly expressed in the liver, and indoleamine-2,3-dioxygenase-1 (IDO1), which is expressed in extrahepatic tissues. Kidney plays an important role in tryptophan metabolism (Hui et al., 2023) and IDO1 is expressed robustly in this tissue (Kanishka et al., 2008). The disturbance in tryptophan metabolism and activation of the kynurenine pathway could cause insulin resistance and lead to the pathogenesis of DN (Yu et al., 2021). It should be emphasized that the chronic kidney disease secondary to T2DM is associated with oxidative stress and inflammation which in turn induces IDO1. There is evidence for activated tryptophan metabolism with concomitant rise in kynurenine and kynurenate in type 2 diabetic patients with impaired kidney function (Subrata et al., 2017). The present data showing increased kynurenine levels in kidney and kynurenate levels in urine in *db/db* mice indicate increased activity of IDO1. The diabetes-associated changes in the levels of kynurenine and kynurenate are blunted by α-MT. This indicates involvement of α-MT-mediated inhibition of IDO1.

Prompted by published reports that blockade of mTOR pathway is useful to prevent the progression of DN (Swaroop et al., 2024) and encouraged by our own findings that blockade of SLC6A14 by α-MT suppresses mTOR signaling (Karunakaran et al., 2011), we examined the proteins associated with this pathway in the present study. These experiments did show that mTOR pathway is activated in *db/db* mouse kidneys and that treatment with α-MT suppressed this pathway. However, once we analyzed the metabolomic profiles of the kidney tissues, we realized that the changes we noted in mTOR signaling pathway was not related to the blockade of SLC6A14 by α-MT. A detailed analysis of tryptophan levels and IDO1 metabolites revealed potential involvement of IDO1 inhibition by α-MT as the mediator of the changes in mTOR pathway. The abundance of tryptophan is negatively correlated with the mTOR/apoptosis pathway, and the activity of IDO1 is positively correlated with the mTOR/apoptosis pathway. This connection between IDO1 and mTOR pathway is strongly supported by several studies (Li et al., 2017; Folgiero et al., 2016).

The mTOR pathway plays a pivotal role in various cellular processes, including growth, metabolism, autophagy, and apoptosis (Marafie et al., 2024). Its activity reflects the metabolic state of the cell and is intertwined with diverse metabolic pathways (Marafie et al., 2024). In the context of DN, aberrant activation of the mTOR signaling is a central element in disease progression (Inoki, 2008; Chen et al., 2021). mTOR is highly sensitive to amino acids, growth factors, and energy levels. Under hyperglycemic conditions, mTOR undergoes abnormal activation, triggering a cascade of pathophysiological responses such as accelerated protein synthesis, enhanced cell growth, and induction of cellular hypertrophy. These events culminate in thickening of the glomerular basement membrane, expansion of mesangial cells, and abnormal accumulation of extracellular matrix components, all of which are hallmark features of DN. In our study, a significant upregulation of pmTOR expression and apoptosis was noted in the renal tissue of DN mice and α-MT markedly suppressed pmTOR and apoptosis. This finding resonates with our metabolomics data, demonstrating that α-MT significantly improved amino acid, energy, membrane, and nicotinamide metabolism. Therefore, we speculate that α-MT exerts its protective effects against DN by inhibiting IDO1, reprogramming amino acid metabolism, and modulating the mTOR/apoptosis pathway.

Another signaling pathway that is relevant to DN involves the nuclear receptor arylhydrocarbon receptor (AhR) (Zhao et al., 2019). The endogenous agonist for this receptor is kynurenine, the product of IDO1 activity on tryptophan. Our metabolomic analysis shows that IDO1 is activated in *db/db* mouse kidneys and α-MT reverses this effect. The decrease in kynurenine levels in α-MT-treated *db/db* mice is an indication of IDO1 inhibition. These changes in IDO1 activity and kynurenine levels are expected to suppress AhR signaling. AhR has profound impact on inflammation and redox status in cells and inhibition of this signaling pathway is known to have beneficial effects in treating chronic kidney diseases, including DN (Zhao et al., 2019). Even though we have not yet interrogated the involvement of the AhR signaling pathway directly in the present study, the decrease in kynurenine levels as a consequence of α-MT treatment strongly suggests suppression of AhR signaling as at least one of the components that underlie the observed beneficial effects in *db/db* mice in terms of DN. Of course, further research will be necessary to validate this conclusion.

In summary, α-MT is effective in protecting against DN. Metabolomics offers valuable insights into the molecular mechanisms underlying this protective effect. Specifically, the modulation of IDO1 activity, amelioration of metabolic dysfunctions, and consequent suppression of mTOR signaling and cellular apoptosis represent key biochemical alterations contributing to the protective action of α-MT against DN. However, there are limitations in this study. The experimental approach used in the present study is almost entirely based on metabolomics. We tried to relate the changes in the metabolomics profiles seen in *db/db* mice treated with α-MT to the known pharmacological effects of α-MT on SLC6A14 and IDO1. There may be additional effects of this tryptophan derivative not yet known that might have contributed to the observed beneficial effects of α-MT in *db/db* mice. To some extent, we were able to provide direct experimental support for the changes in α-MT-mediated mTOR pathway and for the connection of IDO1 inhibition to this phenomenon. In contrast, our conclusions with regard to AhR pathway and its connection to the observed changes in IDO1 mediated by α-MT remain only speculative at present. Further studies are needed to directly determine if AhR signaling is altered in α-MT-treated *db/db* mice and whether such changes contribute to any extent to the observed beneficial effects of α-MT in ameliorating the detrimental features of DN.

## Data Availability

The raw data supporting the conclusions of this article will be made available by the authors, without undue reservation.
