# Supplementary material for: α-methyltryptophan-mediated protection against diabetic nephropathy in db/db mice as studied with a metabolomics approach
Source: Front Pharmacol. 2025 Jan 20;15:1463673. doi: 10.3389/fphar.2024.1463673 (PMC11788373; doi:10.3389/fphar.2024.1463673)
Supplement: Supplementary file 1 [file DataSheet1.docx]

***Supplementary Material***





**Figure S1.** Typical 600 MHz ^1^H NMR spectra for metabolites in kidney (A) and urine (B) in *db*/*db* mice. UK, Unknown metabolites; GPC, Glycerophosphocholine.


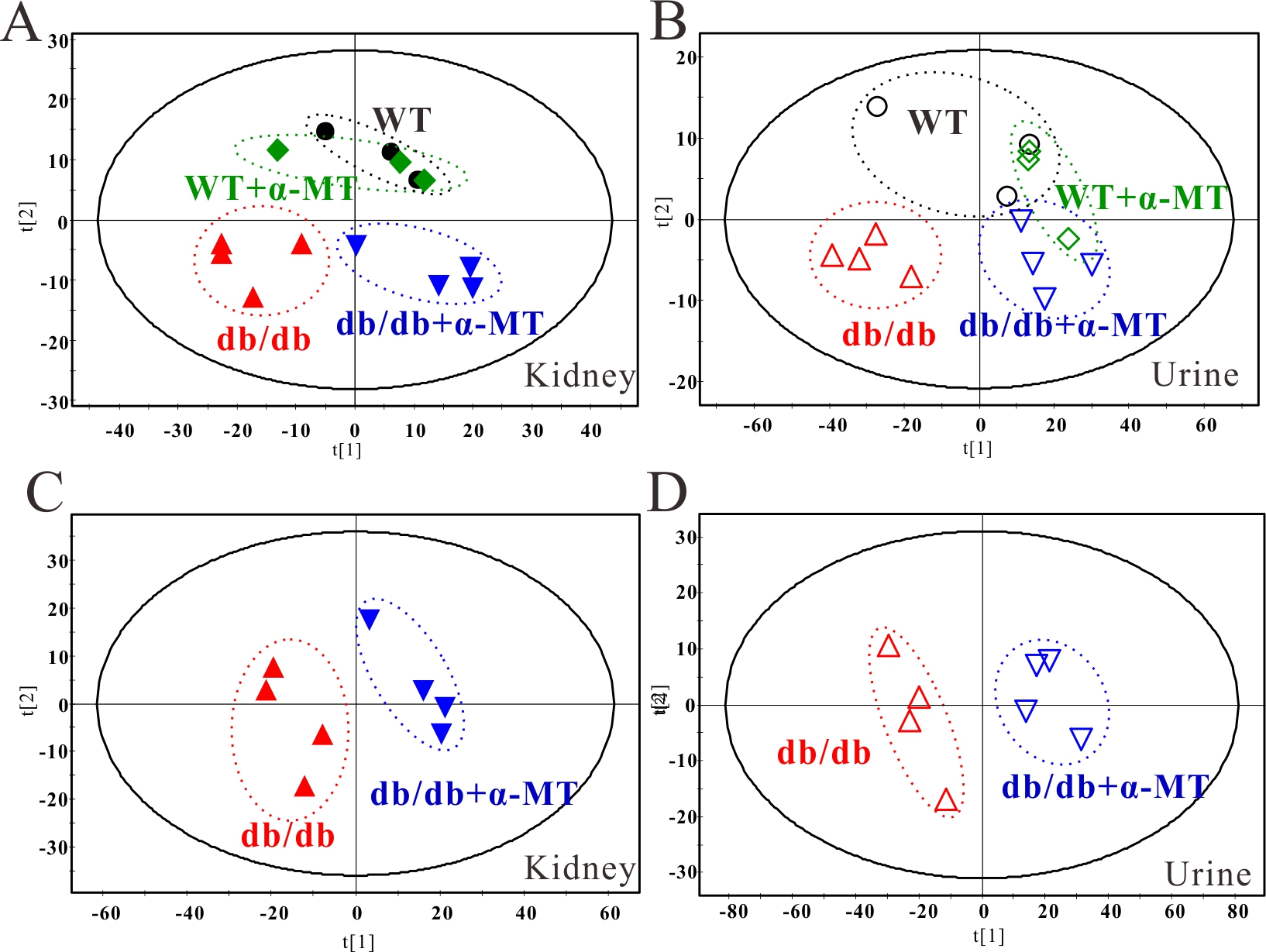


**Figure S2.** Changes of metabolic phenotypes in kidney and urine samples of WT mice and *db*/*db* mice treated with α-MT. PLS-DA score plot was used to identify the variations in the kidney metabolomic profile (A) and in the urine metabolomic profile (B) among four groups. PLS-DA score in the kidney metabolomic profile (C) and in the urine metabolomic profile (D) between control *db*/*db* mice and α-MT-treated *db*/*db* mice.
